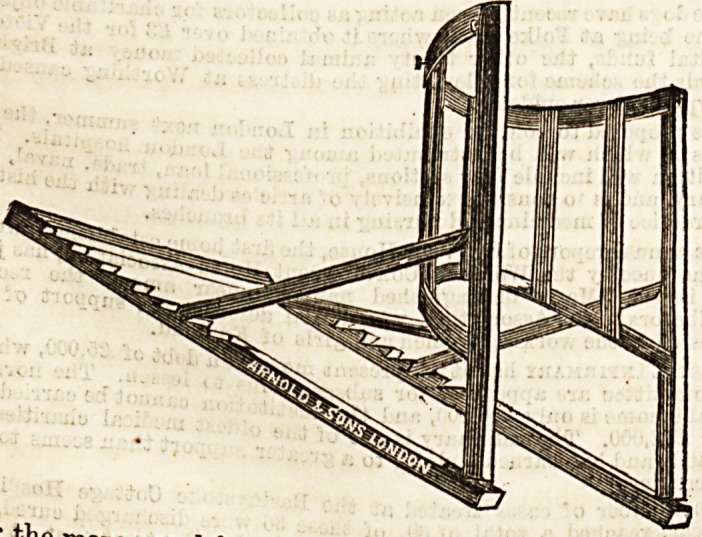# New Bed Rest

**Published:** 1893-10-07

**Authors:** 


NEW BED REST.

The bed-rest of which we give an illustration below, is the invention of Mrs. M. A. Core. It has various advantages over the more usual form of bed-rest, its curved back being a point in its favour; and it can be recommended also on sanitary grounds, as the construction being entirely of wood admits of very complete carbolisation. As will be seen in the drawing, it is specially made to support that part of the back where weakness is most felt, leaving the lower part of the spine, where the skin is most delicate, to rest solely on the soft pillows. A practical test, however, shows that greater comfort would, on the whole, be assured to the patient if another bar were supplied lower down. By means of the notched framework support may be given at any angle, and can be adjusted either to an upright or an almost recumbent position, but in our opinion it would be better with this part of the framework a trifle shorter, about one notch less. At the back of the top bar brass buttons are fixed, to enable a secure arrangement of pillows to be effected with the help of some strong tape, provided with buttonholes. In cases of long and tedious convalescence some such plan will prove very helpful. A couple of pillows, placed lengthwise, with a third across the top as a support for the head, will be found comfortable. The bed-rest is made solely by Messrs. Arnold and Sons, of West Smithfield, to whom we are indebted for the accompanying sketch.

## Figures and Tables

**Figure f1:**